# Impact of a single eGFR and eGFR-estimating equation on chronic kidney disease reclassification: a cohort study in primary care

**DOI:** 10.3399/bjgp18X697937

**Published:** 2018-07-03

**Authors:** Jennifer A Hirst, Maria DLA Vazquez Montes, Clare J Taylor, José M Ordóñez-Mena, Emma Ogburn, Vanshika Sharma, Brian Shine, Tim James, FD Richard Hobbs

**Affiliations:** Nuffield Department of Primary Care Health Sciences, Oxford.; Nuffield Department of Primary Care Health Sciences, Oxford.; Nuffield Department of Primary Care Health Sciences, Oxford.; Nuffield Department of Primary Care Health Sciences, Oxford.; Nuffield Department of Primary Care Health Sciences, Oxford.; Nuffield Department of Primary Care Health Sciences, Oxford.; Oxford University Hospitals Biochemistry Department, John Radcliffe Hospital, Oxford.; Oxford University Hospitals Biochemistry Department, John Radcliffe Hospital, Oxford.; Nuffield Department of Primary Care Health Sciences, Oxford.

**Keywords:** chronic kidney diseases, CKD-EPI, eGFR, glomerular filtration rate, kidney failure, chronic, MDRD, overdiagnosis, renal insufficiency, chronic

## Abstract

**Background:**

Chronic kidney disease (CKD) is diagnosed using the estimated glomerular filtration rate (eGFR) and the urinary albumin:creatinine ratio (ACR). The eGFR is calculated from serum creatinine levels using the Modification of Diet in Renal Disease (MDRD) or Chronic Kidney Disease Epidemiology Collaboration (CKD-EPI) equations.

**Aim:**

To compare the performance of one versus two eGFR/ACR measurements, and the impact of equation choice, on CKD diagnosis and classification.

**Design and setting:**

Cohort study in primary care in the Thames Valley region of the UK.

**Method:**

Data were from 485 participants aged >60 years in the Oxford Renal Cohort Study with at least two eGFR tests. The proportion of study participants diagnosed and classified into different CKD stages using one and two positive tests were compared. Prevalence of CKD diagnosis and classification by CKD stage were compared when eGFR was calculated using MDRD and CKD-EPI equations.

**Results:**

Participants included in the analysis had a mean age of 72.1 (±6.8) years and 57.0% were female. Use of a single screening test overestimated the proportion of people with CKD by around 25% no matter which equation was used, compared with the use of two tests. The mean eGFR was 1.4 ml/min/1.73 m^2^ (95% CI = 1.1 to 1.6) higher using the CKD-EPI equation compared with the MDRD equation. More patients were diagnosed with CKD when using the MDRD equation, compared with the CKD-EPI equation, once (64% versus 63%, respectively) and twice (39% versus 38%, respectively), and 16 individuals, all of who had CKD stages 2 or 3A with MDRD, were reclassified as having a normal urinary ACR when using the CKD-EPI equation.

**Conclusion:**

Current guidance to use two eGFR measures to diagnose CKD remains appropriate in an older primary care population to avoid overdiagnosis. A change from MDRD to CKD-EPI equation could result in one in 12 patients with a CKD diagnosis with MDRD no longer having a diagnosis of CKD.

## INTRODUCTION

Chronic kidney disease (CKD) is a global health problem associated with high levels of morbidity and mortality.^1^^–^3 The prevalence of CKD is increasing worldwide,^4^ with current estimates being approximately 13% in the general population.^5^ There is an age-related decline in renal function, with the largest burden in those aged >60 years.^6^^–^8

CKD is diagnosed using measures of kidney damage or function, including the increased urinary albumin:creatinine ratio (ACR) or decreased glomerular filtration rate (GFR), usually estimated from serum creatinine levels. The Chronic Kidney Disease Epidemiology Collaboration (CKD-EPI) equation is the global standard for estimating GFR, although previously the Modification of Diet in Renal Disease (MDRD) equation was most commonly used.^9^^,^^10^ A description of both equations is available from the authors on request. Adoption of the CKD-EPI equation in the UK was recommended by the National Institute for Health and Care Excellence in 2014^10^ and the target of nationwide roll-out by April 2017 was set by the Association for Clinical Biochemistry and Laboratory Medicine in 2016.^11^ Despite this, many laboratories have not yet converted, and still use, the MDRD equation to test for CKD.

CKD is diagnosed if urinary ACR is ≥3 mg/mmol or the estimated glomerular filtration rate (eGFR) is <60 ml/min/1.73 m^2^ on two occasions at least 90 days apart.^10^^,^^12^ Stages of CKD range from stage 1 in those with normal eGFR but in whom renal damage is present (indicated by an elevated ACR), to stage 5, which constitutes kidney failure. (The thresholds of each CKD stage are available from the authors on request.)^10^^,^^12^

Prevalence estimates of CKD can vary widely, depending on whether they are based on a single eGFR (which may result in a false positive rate of 30–50%) or two tests (as recommended by guidelines set by Kidney Disease: Improving Global Outcomes [KDIGO]).^13^^,^^14^ Furthermore, introduction of the CKD-EPI equation may result in reduced prevalence of CKD;^13^ however, despite CKD often being managed by primary care, data from primary care populations is lacking.

This study reports findings from an analysis of data from the Oxford Renal Cohort Study (OxRen),^15^ which recruited 3205 participants aged >60 years from primary care within the Thames Valley region of the UK. It details how CKD prevalence estimates and stages of CKD differ, depending on whether a single eGFR or two measures (as recommended by KDIGO guidelines) are used, and compares proportions of patients classified in each CKD stage when the CKD-EPI equation versus the MDRD equation is used.

**How this fits in**

Diagnosis of chronic kidney disease (CKD) is based on two screening tests; reports have suggested that a single test could overestimate the prevalence of CKD by as much as 50%. This study has found that a single test overestimated CKD by 25% in an older primary care population, suggesting that a second screening test is clinically appropriate. As laboratories move from using the Modification of Diet in Renal Disease (MDRD) equation to the Chronic Kidney Disease Epidemiology Collaboration (CKD-EPI) equation to estimate glomerular filtration rate, some people will no longer have a CKD diagnosis. This study found that 8% of people with a CKD diagnosis based on use of the MDRD equation will be reclassified as not having CKD if the CKD-EPI equation is used.

## METHOD

Data were used from 485 participants in the OxRen study who had two CKD screening tests, which had been conducted between 90 days and 2 years (730 days) apart. The remainder of study participants had either a single screening test or the second test fell outside the time limits. All samples were analysed across two laboratories using identical ACR and isotope dilution mass spectrometry (IDMS)-traceable creatinine assays, and reported the MDRD eGFR. Both samples for each patient were processed in the same laboratory.

The MDRD eGFR, patient age (at each screening test), sex, and ethnicity were used to calculate the CKD-EPI eGFR. The mean patient age was used for those for whom age was missing (*n* = 30, 6.2%) and, as the recruitment area for OxRen has a majority white population, white ethnicity was allocated to 30 patients (6.2%) for whom no ethnicity was recorded. Those for whom no data regarding their sex were recorded (*n* = 9, 1.9%) were excluded. People with missing ACR results (*n* = 8 [1.6%], and *n* = 6 [1.2%] in first and second screening test respectively) were included in the analysis, and the ACR was assumed to be >3 mg/mmol. The majority of participants included in this cohort had two screening tests because the first screening test suggested a positive CKD diagnosis. On this basis the higher ACR was used. The same assumptions were made for the first and second tests. Results were compared with eGFR results calculated directly from serum creatinine levels in a subset of patients for whom this was available. Sensitivity analyses were carried out to explore the impact of these approximations.

Numbers of patients diagnosed with CKD, sex, age, ethnicity, and mean and standard deviation (SD) time between screening visits were tabulated. The mean and SDs of the eGFR calculated using the MDRD and CKD-EPI equations were summarised by using the first measure alone, the second measure alone, and the mean of both measures. Proportions of patients diagnosed with CKD using a single eGFR/ACR screening test and two positive eGFR/ACR screening tests using the MDRD and CKD-EPI equations were tabulated.

Using both equations, the numbers of patients classified into each CKD stage using a single test and two eGFR measures (the second of two positive measures, the higher of two positive eGFRs, and the mean of two positive eGFRs) were calculated. Results were tabulated to:

- show how patients were reclassified into different CKD stages using both the MDRD and CKD-EPI equations and two measures, instead of one, were used to calculate eGFRs; and
- compare diagnoses and reclassification of CKD stage when the CKD-EPI equation is used instead of the MDRD equation.

Scatter plots were used to compare the first and second eGFR measures for both equations. Bland–Altman plots^16^ were used to compare the mean difference between the first and second measures with the mean of both measures. The eGFR, calculated using the CKD-EPI and MDRD equations for the first and second measure for each patient, were plotted to show agreement between equations. A multinomial logistic regression analysis was used to examine the association between age, sex, and change in CKD stage when moving from the MDRD to the CKD-EPI equation. All analyses were carried out using StataCorp’s Stata/SE version 14.

## RESULTS

Participants’ mean age was 72.1 (±6.8) years, 57.5% were female, and 98.9% of those with data had white ethnicity. The mean time between screening tests was 153 (±107) days (range 90–728 days) (Table 1).

**Table 1.** Description of cohort, *n* = 485

**Descriptor**	**Data**
**Age in years at first test**	
Mean (SD)	72.1 (6.8)
Range	60.0–93.2

**Sex**	
Female, %	57.5

**Time between tests in days**	
Mean (SD)	153 (107)
Median (IQR)	112.5 (100–147)

**Ethnicity, *n***	
White	449
Mixed ethnicity	4
Black	1

*IQR* = *interquartile range. SD* = *standard deviation.*

When using the second measure alone, the mean eGFR generated when using the CKD-EPI equation was 1.4 ml/min/1.73 m^2^ (95% confidence interval [CI] = 1.1 to 1.6) higher than that generated when using the MDRD equation: 68.3 and 66.9ml/min/1.73m^2^ for CKD-EPI and MDRD respectively (Table 2).

**Table 2.** Mean eGFR and CKD diagnosis based on single or duplicate measures using the MDRD and CKD-EPI equations

	**MDRD equation, *n*= 485**	**CKD-EPI equation, *n*= 476^a^**
eGFR (ml/min/1.73 m^2^) using first measure, mean (SD)	66.4 (12.2)	67.5 (12.1)
eGFR (ml/min/1.73 m^2^) using second measure, mean (SD)	66.9 (11.9)	68.3 (12.2)
eGFR (ml/min/1.73 m^2^) using both measures, mean (SD)	66.6 (12.1)	67.9 (12.0)
Classified as having CKD based on first eGFR alone, *n* (%)	311 (64)	301 (63)
Classified as having CKD based on two positive eGFRs, *n* (%)	190 (39)	182 (38)
Incorrectly classified using a single measure, *n* (%)	121 (25)	119 (25)

a *Nine patients excluded as sex missing. CKD* = *chronic kidney disease. CKD-EPI* = *Chronic Kidney Disease Epidemiology Collaboration. eGFR* = *estimated glomerular filtration rate. MDRD* = *Modification of Diet in Renal Disease. SD* = *standard deviation.*

The number of patients diagnosed with CKD using each equation and a single test or two positive eGFR/ACR measures are shown in Table 2. The MDRD and CKD-EPI equations classify similar numbers of patients as having CKD whether the first eGFR alone or two measures are used: 64% of patients with the MDRD equation versus 63% using the CKD-EPI equation for a single screening test, and 39% (MDRD equation) and 38% (CKD-EPI equation) when using two positive tests. Both equations incorrectly classified approximately 25% using a single test. Results were similar in sensitivity analyses using only those with complete data (available from the authors on request) and laboratory-reported serum creatinine levels (data not shown).

The number of participants classified into different CKD stages using a single and two positive eGFR/ACR measures for both equations are shown in Table 3. Using the second of two positive measures, fewer patients were classified as having stage 1 CKD and more as having stage 2 CKD with the CKD-EPI equation than with the MDRD equation (2% versus 7% and 50% versus 45% respectively). Using the CKD-EPI and the MDRD equations, similar proportions of patients were classified as having stage 3A CKD (40% versus 41%) and stage 3B CKD (8% versus 7% respectively).

**Table 3.** Patients categorised by CKD stage using a single and two eGFR measures for the MDRD and CKD-EPI equations

**CKD stage**	**MDRD equation**				**CKD-EPI equation**			
	**Single eGFR (*n*= 311), *n* (%)**	**Two eGFRs, *n*= 190**			**Single eGFR (*n*= 301), *n* (%)**	**Two eGFRs, *n*= 182**		
		**Second eGFR, *n* (%)**	**Highest eGFR, *n* (%)**	**Mean of both eGFRs, *n* (%)**		**Second eGFR, *n* (%)**	**Highest eGFR, *n* (%)**	**Mean of both eGFRs, *n* (%)**
Stage 1	41 (13)	14 (7)	20 (11)	10 (5)	19 (6)	4 (2)	7 (4)	5 (3)
Stage 2	142 (46)	85 (45)	91 (48)	96 (51)	166 (55)	91 (50)	100 (55)	98 (54)
Stage 3A	115 (37)	77 (41)	70 (37)	71 (37)	102 (34)	72 (40)	65 (36)	65 (36)
Stage 3B	13 (4)	14 (7)	9 (5)	13 (7)	13 (4)	15 (8)	10 (5)	14 (8)
Stage 4	–	–	–	–	1 (0)	–	–	–
Stage 5	–	–	–	–	–	–	–	–

*CKD* = *chronic kidney disease. CKD-EPI* = *Chronic Kidney Disease Epidemiology Collaboration. eGFR* = *estimated glomerular filtration rate. MDRD* = *Modification of Diet in Renal Disease.*

Results for reclassification of CKD stage from a single to two positive eGFRs (using the second value) for both equations are presented in Table 4. The proportion of patients who remained in the same category with two measures increased as the CKD stage of the first test increased for both equations; fewer people were reclassified as having normal urinary ACR if the first test indicated stage 3B CKD. Overall, using the MDRD equation and two eGFR/ACR measures instead of one resulted in 137 people (44%) moving to a lower CKD stage or being classified as having a normal urinary ACR, 22 (7%) moving to a higher CKD stage, and 152 (49%) remaining in the same CKD stage. When using the CKD-EPI equation and the second of two positive measures instead of a single eGFR, 127 people (42%) moved to a lower CKD stage or were reclassified as having a normal urinary ACR, 19 (6%) moved to a higher CKD stage, and 155 (51%) remained diagnosed with the same CKD stage.

**Table 4.** Reclassification of patients’ CKD stage when moving from a single test to the second of two eGFR measures^a^

**CKD stage at first test**	**MDRD equation, *n*= 311**						**CKD-EPI equation, *n*= 301**					
	**First, eGFR *n*= 311, *n***	**CKD stage at follow-up eGFR**					**First eGFR, *n*= 301, *n* (%)**	**CKD stage at follow-up eGFR**				
		**Normal urinary ACR, *n*= 121, *n* (%)**	**Stage 1, *n*= 14, *n* (%)**	**Stage 2, *n*= 85, *n* (%)**	**Stage 3A, *n*= 77, *n* (%)**	**Stage 3B, *n*= 14, *n* (%)**		**Normal urinary ACR, *n*= 119, *n* (%)**	**Stage 1, *n*= 4, *n* (%)**	**Stage 2, *n*= 91, *n* (%)**	**Stage 3A, *n*= 72, *n* (%)**	**Stage 3B, *n*= 15, *n* (%)**
**Stage 1**	41	25 (61)	10^b^ (24)	6 (15)	0 (0)	0 (0)	19	12 (63)	4^b^ (21)	3 (16)	0 (0)	0 (0)
**Stage 2**	142	56 (39)	4 (3)	70^b^ (49)	11 (8)	1 (0)	166	71 (43)	0 (0)	83^b^ (50)	11 (7)	1 (0)
**Stage 3A**	115	39 (34)	0 (0)	9 (8)	63^b^ (55)	4 (3)	102	35 (34)	0 (0)	5 (5)	58^b^ (57)	4 (4)
**Stage 3B**	13	1 (7)	0 (0)	0 (0)	3 (23)	9^b^ (69)	13^c^	1 (8)	0 (0)	0 (0)	3 (23)	9^b^ (69)

a Percentages are presented across rows representing those reclassified after the second measure for each stage of CKD, and separately for MDRD and CKD-EPI.

b Numbers of patients for whom the CKD stage did not change.

c One patient was Stage 4 at the first test and stage 3B (100%) after the second test. ACR = albumin:creatinine ratio. CKD = chronic kidney disease. CKD-EPI = Chronic Kidney Disease Epidemiology Collaboration. eGFR = estimated glomerular filtration rate. MDRD = Modification of Diet in Renal Disease.

The numbers of participants classified into different CKD stages when the eGFR was calculated using the CKD-EPI instead of the MDRD equation are shown in Table 5. When the CKD-EPI equation was used, the classified CKD stage remained the same for the majority of individuals; however, 18 of those diagnosed as having CKD when measured using the MDRD equation moved to a lower CKD stage or were classified as having normal kidney function, and 15 were diagnosed with a higher stage of CKD. Overall, 17% of those classified into stages 1–3B using the MDRD equation were reclassified when using the CKD-EPI equation — this included 14 patients who were reclassified from stage 3A to normal kidney function (non-CKD) and eight patients who were reclassified from having normal kidney function to having stage 3A CKD.

**Table 5.** Reclassification of CKD stage when moving from the MDRD to the CKD-EPI equation using the second eGFR/ACR result after a positive first screening, *n* = 476^a^

**CKD stage based on MDRD equation**	***n***	**CKD stage based on CKD-EPI equation**				
		**Normal urinary ACR, *n*= 294, *n***	**Stage 1, *n*= 4, *n***	**Stage 2, n = 91, *n***	**Stage 3A, *n*= 72, *n***	**Stage 3B, *n*= 15, *n***
**Normal urinary ACR**	286	278^b^	0	0	8	0
**Stage 1**	14	0	4^b^	10	0	0
**Stage 2**	85	2	0	79^b^	4	0
**Stage 3A**	77	14	0	2	60^b^	1
**Stage 3B**	14	0	0	0	0	14^b^

a Nine patients excluded as sex missing.

b *Numbers of patients for whom the CKD stage did not change. ACR* = *albumin:creatinine ratio. CKD* = *chronic kidney disease. CKD-EPI* = *Chronic Kidney Disease Epidemiology Collaboration. eGFR* = *estimated glomerular filtration rate. MDRD* = *Modification of Diet in Renal Disease.*

**Figure 1.**
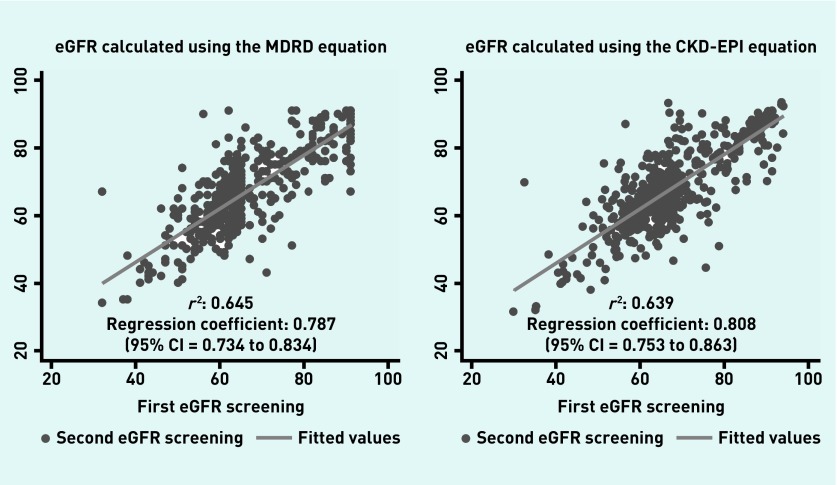
***The second eGFR plotted against the first eGFR measures estimated using the MDRD and CKD-EPI equations. CKD-EPI = Chronic Kidney Disease Epidemiology Collaboration. eGFR = estimated glomerular filtration rate. MDRD = Modification of Diet in Renal Disease.***

Correlation plots between the first and second eGFR measures for both equations are shown in Figure 1. The regression analysis showed similar agreement between the first and second measures using the MDRD equation (*r*^2^ = 0.645) compared with the CKD-EPI equation (*r*^2^ = 0.639). Bland–Altman plots showed good agreement between the mean difference in the eGFR between the first and second measurement versus the mean of two measurements for the MDRD and CKD-EPI equations. On average, the first measure was higher than the second measure for each equation (data available from the authors on request). The CKD-EPI eGFR plotted against the MDRD eGFR for the first and second eGFR measures showed overall agreement (data available from the authors on request).

The multinomial logistic regression analysis found a statistically significant association between age and reclassification of CKD stage when moving from the MDRD to the CKD-EPI equation (data not shown). An increase of 1 year in age is associated with a 7% lower risk of being reclassified to a lower CKD stage with the CKD-EPI equation compared with the CKD stage remaining the same (relative risk ratio [RRR] 0.93, 95% CI = 0.85 to 1.01), and a 12% higher risk of being reclassified to a higher CKD stage (RRR 1.12, 95% CI = 1.06 to 1.19). Multinomial logistic regression directly compared those with an increase in CKD stage with those with a decrease in CKD stage. This confirmed that participants were 20% more likely to be reclassified to a higher stage using the CKD-EPI equation for each increase in year of age than they were to be reclassified to a lower stage, compared with no change in CKD stage between equations.

Males were 3.99 times (95% CI = 1.50 to 10.59) more likely to be reclassified to a higher CKD stage than females when the CKI-EPI equation was used instead of the MDRD equation (data not shown).

## DISCUSSION

### Summary

This analysis has found that, in an older primary care population, a single CKD screening test overestimates the number of people diagnosed with CKD by around 25%, compared with two tests. The mean eGFR was higher using the CKD-EPI equation compared with the MDRD equation, indicating that fewer patients will have a diagnosis of CKD when laboratories move to reporting eGFR using the CKD-EPI equation.

Most people in the cohort had stage 2 CKD, followed by those with stage 1 CKD, indicating that they had an abnormal ACR result but normal or mildly reduced eGFR. These people would retain their CKD diagnosis regardless of the equation used. Most participants whose first CKD screening test indicated a CKD diagnosis that was not confirmed in the follow-up test were, using the first test alone, initially classified as having stage 1–3A CKD; this suggests that the reclassification stems from variability in the ACR as well as serum creatinine levels. Reclassification was most marked for those with stage 3A CKD using the MDRD equation; in total, 18% of this group were reclassified as having normal urinary ACR using the CKD-EPI equation (Table 5) — all had normal ACR results and the diagnosis was based solely on reduced MDRD eGFR. Prevalence of CKD was 39% in this pre-selected cohort with two ACR/eGFR tests.

### Strengths and limitations

To the authors’ knowledge, this is the largest analysis to compare CKD diagnoses and reclassification, based on single and twice-conducted screening tests in an older population in primary care. The study presented here is unique, as far as the authors are aware, in reporting results from an older primary care population. However, in carrying out this analysis, it was only possible to access eGFRs that had been calculated using the MDRD equation. Using the available data on age and ethnicity, the authors were able to calculate the eGFR using the CKD-EPI equation, but some assumptions had to be made to use the full dataset. The impact of these estimations have been assessed in a sub-sample of patients with creatinine results and in a sensitivity analysis, which showed similar results.

A further limitation is that the cohort was mainly of white ethnicity and so may not be generalisable to the general UK population.

It is acknowledged that both equations used to calculate the eGFR in this analysis exhibit some bias relative to measuring GFR;^17^ results may differ if the GFR is measured using gold-standard methods, which involves using radioisotope or iothalamate methods and taking multiple blood or urine samples.^18^

### Comparison with existing literature

Previous studies have suggested that the prevalence of CKD may be overestimated by 30–50% using a single eGFR compared with two positive measures.^13^^,^^14^^,^^19^ However, not all studies used the KDIGO criteria for diagnosis,^8^ or studied populations with low CKD prevalence.^14^^,^^20^ Prevalence of CKD was 39% in this pre-selected cohort with two ACR/eGFR tests.

The finding presented here, that the CKD-EPI equation gave a higher mean eGFR and reduced the prevalence of CKD compared with the MDRD equation, has been reported by other researchers.^7^^,^^13^^,^^21^^,^^22^ In this study, it was found that 17% of patients were reclassified using the CKD-EPI rather than the MDRD equation; this is similar to numbers from a previous report,^23^ in which reclassification to a lower CKD stage was associated with fewer adverse outcomes.

Previous research has found that using the CKD-EPI equation increased CKD prevalence compared with use of the MDRD equation;^24^ this may be an artefact of participant age and prevalence or severity of CKD in the populations studied, or the relative accuracy of the creatinine method. Using the CKD-EPI equation instead of the MDRD equation may decrease the proportion of younger patients (<60 years) diagnosed with CKD, but the reverse may be true in some older patient groups.^7^^,^^22^ The regression analysis presented here found that older age and male sex were associated with reclassification to a higher CKD stage using the CKD-EPI equation.

A recent meta-analysis has shown that the CKD-EPI equation gives a better eGFR than the MDRD equation, particularly in patients with the highest kidney function.^17^ Although the age of the OxRen cohort was >60 years, none of the patients had severely decreased kidney function in whom the MDRD equation may perform better.^7^^,^^17^

The strong association between reclassification of CKD stage in males may be due to the effect of muscle mass on creatinine-based measures,^25^ which could be avoided with cystatin C-based equations.^25^^,^^26^

### Implications for practice

This study has found that a single eGFR/ACR test would result in an unacceptably high rate of overdiagnosis of CKD, and confirms that current recommendations to base CKD diagnoses on two positive tests — thereby allowing for biological and analytical variability — are appropriate in primary care.

As laboratories move from using the MDRD to the CKD-EPI equation, further consideration is needed to determine how those patients who had a CKD diagnosis but fall below the CKD threshold with the new equation should be treated and followed up as there is no guidance on how GPs should respond or how this could be communicated to patients. If these patients retain their CKD diagnoses, despite no longer meeting the diagnostic criteria, they may receive unnecessary monitoring, treatment, and interventions.

Fewer than 1% of patients with stage 3A CKD are reported to go on to develop end-stage renal disease during 8 years of follow-up^27^ and there have been suggestions that definitions of CKD may need to change, particularly for those people who have stage 3A CKD without albuminuria.^28^ Although kidney function declines with age and decreasing eGFR is associated with increased mortality,^29^ many older people are diagnosed with CKD without being at high risk of adverse outcomes; it has, therefore, been suggested that using different cut-offs for older people may be appropriate.^30^ On this basis, it is likely that the move to the CKD-EPI equation will result in more-appropriate CKD classification based on the risk of future adverse outcomes^23^ and comparison with measured GFR^17^ for this population.

The time interval between tests may also affect the agreement between the tests. KDIGO defines chronic CKD as a duration >3 months, but this time interval was selected arbitrarily.^12^ If the minimum length of time between CKD screenings tests is extended to 12 months, the prevalence of CKD stages 3–4 could decrease by 37%;^31^ this may have implications for the definition of chronicity of CKD in future. In the analysis presented here, the KDIGO definition of a minimum of 3 months has been accepted, but some tests were up to 2 years apart. On this basis, this study has found that current guidance to use two eGFR measures to diagnose CKD remains appropriate in an older primary care population to avoid overdiagnosis.
